# Regularized Machine Learning in the Genetic Prediction of Complex Traits

**DOI:** 10.1371/journal.pgen.1004754

**Published:** 2014-11-13

**Authors:** Sebastian Okser, Tapio Pahikkala, Antti Airola, Tapio Salakoski, Samuli Ripatti, Tero Aittokallio

**Affiliations:** 1 Department of Information Technology, University of Turku, Turku, Finland; 2 Turku Centre for Computer Science (TUCS), University of Turku and Åbo Akademi University, Turku, Finland; 3 Hjelt Institute, University of Helsinki, Helsinki, Finland; 4 Institute for Molecular Medicine Finland (FIMM), University of Helsinki, Helsinki, Finland; 5 Wellcome Trust Sanger Institute, Hinxton, United Kingdom; University of California San Diego and The Scripps Research Institute, United States of America

## Overview

Compared to univariate analysis of genome-wide association (GWA) studies, machine learning–based models have been shown to provide improved means of learning such multilocus panels of genetic variants and their interactions that are most predictive of complex phenotypic traits. Many applications of predictive modeling rely on effective variable selection, often implemented through model regularization, which penalizes the model complexity and enables predictions in individuals outside of the training dataset. However, the different regularization approaches may also lead to considerable differences, especially in the number of genetic variants needed for maximal predictive accuracy, as illustrated here in examples from both disease classification and quantitative trait prediction. We also highlight the potential pitfalls of the regularized machine learning models, related to issues such as model overfitting to the training data, which may lead to over-optimistic prediction results, as well as identifiability of the predictive variants, which is important in many medical applications. While genetic risk prediction for human diseases is used as a motivating use case, we argue that these models are also widely applicable in nonhuman applications, such as animal and plant breeding, where accurate genotype-to-phenotype modeling is needed. Finally, we discuss some key future advances, open questions and challenges in this developing field, when moving toward low-frequency variants and cross-phenotype interactions.

## Introduction

Supervised machine learning aims at constructing a genotype–phenotype model by learning such genetic patterns from a labeled set of training examples that will also provide accurate phenotypic predictions in new cases with similar genetic background. Such predictive models are increasingly being applied to the mining of panels of genetic variants, environmental, or other nongenetic factors in the prediction of various complex traits and disease phenotypes [1]–[8]. These studies are providing increasing evidence in support of the idea that machine learning provides a complementary view into the analysis of high-dimensional genetic datasets as compared to standard statistical association testing approaches. In contrast to identifying variants explaining most of the phenotypic variation at the population level, supervised machine learning models aim to maximize the predictive (or generalization) power at the level of individuals, hence providing exciting opportunities for e.g., individualized risk prediction based on personal genetic profiles [9]–[11]. Machine learning models can also deal with genetic interactions, which are known to play an important role in the development and treatment of many complex diseases [12]–[16], but are often missed by single-locus association tests [17]. Even in the absence of significant single-loci marginal effects, multilocus panels from distinct molecular pathways may provide synergistic contribution to the prediction power, thereby revealing part of such *hidden heritability* component that has remained missing because of too small marginal effects to pass the stringent genome-wide significance filters [18]. Multivariate modeling approaches have already been shown to provide improved insights into genetic mechanisms and the interaction networks behind many complex traits, including atherosclerosis, coronary heart disease, and lipid levels, which would have gone undetected using the standard univariate modeling [2], [19]–[22]. However, machine learning models also come with inherent pitfalls, such as increased computational complexity and the risk for model overfitting, which must be understood in order to avoid reporting unrealistic prediction models or over-optimistic prediction results.

We argue here that many medical applications of machine learning models in genetic disease risk prediction rely essentially on two factors: effective model regularization and rigorous model validation. We demonstrate the effects of these factors using representative examples from the literature as well as illustrative case examples. This review is not meant to be a comprehensive survey of all predictive modeling approaches, but we focus on *regularized machine learning models*, which enforces constraints on the complexity of the learned models so that they would ignore irrelevant patterns in the training examples. Simple risk allele counting or other multilocus risk models that do not incorporate any model parameters to be learned are outside the scope of this review; in fact, such simplistic models that assume independent variants may lead to suboptimal prediction performance in the presence of either direct or indirect interactions through epistasis effects or linkage disequilibrium, respectively [23], [24]. Perhaps the simplest models considered here as learning approaches are those based on weighted risk allele summaries [23], [25]. However, even with such basic risk models intended for predictive purposes, it is important to learn the model parameters (e.g., select the variants and determine their weights) based on training data only; otherwise there is a severe risk of *model overfitting*, i.e., models not being capable of generalizing to new samples [5]. Representative examples of how model learning and regularization approaches address the overfitting problem are briefly summarized in Box 1, while those readers interested in their implementation details are referred to the accompanying Text S1. We specifically promote here the use of such regularized machine learning models that are scalable to the entire genome-wide scale, often based on linear models, which are easy to interpret and also enable straightforward variable selection. Genome-scale approaches avoid the need of relying on *two-stage approaches* [26], which apply standard statistical procedures to reduce the number of variants, since such prefiltering may miss predictive interactions across loci and therefore lead to reduced predictive performance [8], [24], [25], [27], [28].

**Box 1. Synthesis of Learning Models for Genetic Risk Prediction**

The aim of risk models is to capture in a mathematical form the patterns in the genetic and non-genetic data most important for the prediction of disease susceptibility. The first step in model building involves choosing the functional form of the model (e.g., linear or nonlinear), and then making use of a given training data to determine the adjustable parameters of the model (e.g., a subset of variants, their weights, and other model parameters). While it is often sufficient for a statistical model to enable high enough explanatory power in the discovery material, without being overly complicated, a predictive model is also required to generalize to unseen cases.

One consideration in the model construction is how to encode the genotypic measurements using genotype models, such as the dominant, recessive, multiplicative, or additive model, each implying different assumptions about the genetic effects in the data [79]. Categorical variables 0, 1, and 2 are typically used for treating genetic predictor variables (e.g., minor allele dosage), while numeric values are required for continuous risk factors (e.g., blood pressure). Expected posterior probabilities of the genotypes can also be used, especially for imputed genotypes. Transforming the genotype categories into three binary features is an alternative way to deal with missing values without imputation (used in the T1D example; see Text S1 for details).

Statistical or machine learning models identify statistical or predictive interactions, respectively, rather than biological interactions between or within variants [12], [80]. While nonlinear models may better capture complex genetic interactions [7], [81], linear models are easier to interpret and provide a scalable option for performing supervised selection of multilocus variant panels at the genome-wide scale [3]. In linear models, genetic interactions are modeled implicitly by selecting such variant combinations that together are predictive of the phenotype, rather than considering pairwise gene–gene relationships explicitly. Formally, trait *y_i_* to be predicted for an individual *i* is modeled as a linear combination of the individual's predictor variables *x_ij_*:
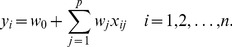
(1)Here, the weights *w_j_* are assumed constant across the *n* individuals, *w*_0_ is the bias offset term and *p* indicates the number of predictors discovered in the training data. In its basic form, Eq. 1 can be used for modeling continuous traits *y* (linear regression). For case-control classification, the binary dependent variable *y* is often transformed using a logistic loss function, which models the probability of a case class given a genotype profile and other risk factor covariates *x* (logistic regression). It has been shown that the logistic regression and naïve Bayes risk models are mathematically very closely related in the context of genetic risk prediction [81].

*Model regularization* refers to the technique of controlling the model complexity, with the aim of preventing overfitting the model to the training data, and hence to improve its generalization capability to new samples. Classical regularization approaches rely on explicit penalization of the model complexity through penalty terms such as *L*_1_ and *L*_2_ norms for model weights (Figure 2A). Together with the squared loss function (Figure 2B), which is often used to measure the fit between the observed 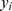 and estimated 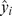 phenotypes (Eq.1), these functional norms give rise to the optimization problem used in various types of linear genetic risk prediction models:
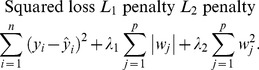
(2)Ridge regression is the special case of Eq. 2, in which 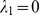, and the regularization parameter 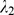 is used to shrink the variable weights toward zero to prevent any particular variable from having too large effect on the model. However, the use of *L*_2_ penalty alone tends to favor models that depend on all the variables. In Lasso, 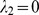, and through adjusting the regularization parameter 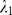, it is possible to favor sparse models with only a few nonzero weights, leading to variable selection within the model fitting [82]. The Elastic Net model makes use of both penalty terms *L*_1_ and *L*_2_ to select also correlated features [83]; for instance, groups of variants within a pathway that together contribute to the predictive accuracy.

Methods such as Lasso and Elastic Net are traditionally known as *embedded models*, since the feature selection is embedded into the learning algorithm itself [5]. These methods select the features simultaneously and therefore do not provide the user with a direct control over the number of variables to be selected in the final prediction model, although heuristics based on absolute weights and other tuning criterion can be used for ranking the variables [24], [84]. In contrast, *wrapper models* enable the user to preset the number of features in the final model. However, due to the exponentially increasing size of the genetic search spaces, in practice one must resort to local search methods, such as greedy feature selection implemented e.g., in *L*_2_-RLS wrappers [30].

The wrapper and embedded methods are not distinct classes of algorithms. Scalable wrappers often incorporate elements of embedded methods to guarantee computational efficacy. For instance, RLS shares similar properties with Lasso and linear variants of SVMs. The accompanying Text S1 describes interrelationships between different learning models in terms of their norms and loss functions (Figure 2), including squared loss (RLS, Lasso and Elastic Net), logistic loss (logistic regression) and hinge loss (SVMs). It also presents a generic optimization framework that implements some of the most efficient methods currently available for genome-wide data. There are also other implementations available, including Mendel [85], HyperLasso [86] and SparSNP [87], gpu-lasso [88], and PUMA [89].

In addition to the classical regularization approaches, where an explicit model complexity penalization term is included in the optimization problem (Eq. 2), alternative strategies have been developed for avoiding overfitting. Among the most popular ones are *ensemble learning*, implemented e.g., in the popular Random Forests (RF) algorithm [90]–[92], as well as in the Bayesian modeling approaches, where probabilistic prior distributions on the model parameters are used for the shrinkage and regularization purposes [93]–[95]. Other approaches are based on the ensemble of models composed of varying number of features [96], bagging or boosting and various search-based algorithms [3]. From the theoretical viewpoint, however, all of these learning approaches can be considered as different types of regularization approaches [97]–[100].

Whereas classical, univariate filter methods evaluate the relevance of each genetic feature independently of the others, more advanced *multivariate filters* have also been proposed, including the Relief family of approaches [101]. The main advantage of the multivariate filters over the univariate ones is that they can detect complex relationships between multiple genetic features and also yield smaller feature sets with less redundancy. Results from the ReliefF runs can also be aggregated, similar to ensemble learning, to yield more robust variant rankings and identification of gene–gene interactions [102]. However, multivariate filters also have specific limitations, such that their selection criteria are not directly connected to the generalization capability of the final prediction model, which may lead to suboptimal results [103].

Even advanced machine learning methodologies have been shown to be negatively affected by the presence of *population stratification*, leading to either false positives or false negative detections. To avoid the need to cluster the data into smaller substrata according to population structures, learning machines can be complemented by information of such substructures extracted using feature extraction methods, such as EIGENSTRAT, PCA, or MDS [104]. Lasso has been extended to account for population structures through linear mixed models [105], which are gaining much popularity in association studies [106]. Machine learning methods enable also the detection of population substructures, for instance, by learning ensembles of decision trees that are capable of accurately predicting individual's subcontinental ancestry [107].

*Linkage disequilibrium* (LD) tends to lead to the selection of highly correlated genetic features when using unpenalized modeling approaches [24]. A simple strategy is to select SNPs in linkage equilibrium, but this cannot distinguish the functionally relevant variants from the nonfunctional ones. Alternative approaches have revised, for instance, the tree-building process or importance measure calculation in RF [108], or replaced the univariate split functions by nonlinear multivariate split functions of contiguous SNPs, modeled as decision trees, to better account for SNP correlations [109]. Penalization strategies, such as ridge regression, Lasso and RLS, allow the model to avoid placing too much weight on potentially overfit variables in the presence of LD, which can lead to improved selection of causal variants [110], [111].

Finally, *whole-genome prediction* (WGP) models fit all of the genotyped variants of the genetic data onto ridge regression type of linear models, such as genomic best linear unbiased prediction (GBLUP) or its variants [34], [112]. WGP approach has been widely used in animal and plant breeding applications [113]–[115] and, with recent improvements, increasingly also in human genetics [116], [117]. However, imperfect LD between markers and the causal loci can impose suboptimal prediction accuracy of WGP, especially when analyzing unrelated individuals, but this can be improved through variable selection or other model regularization approaches [61]. Moreover, due to the lack of direct control for the number of variants, WGP approaches are not optimal for those applications in which the size of the genotyped variant panel is limited.

## Preview: Selection of Genetic Variants into the Predictive Models

A recent perspective article gave an excellent overview of the common concepts and potential pitfalls when making predictions of complex phenotypes using genotypic data [28]; however, one of the key components in the construction of predictive models—variant selection—was ignored in this and many other previous works. In the context of machine learning, a method known as *feature selection* is commonly implemented to identify the subset of variants having most predictive power for the particular phenotypic trait. The aims of feature selection include the reduction of the dimensionality of the genetic search space, excluding correlated variants without independent contribution to the prediction, and facilitating the implementation of the final prediction model, for instance, in clinical setup. Three main types of feature selection methods have traditionally been considered in the context of genetic predictors: filters, wrappers, and embedded methods (Box 1). These methods have different characteristics in terms of their computational complexities, potential to detect joint effects between variants, and whether the feature selection is done explicitly in the optimization process or implicitly through model regularization, which make them more or less suitable for different application cases [5]–[8].

A class of widely used filter approaches includes the standard multilocus genetic risk models, where the risk alleles and their weights are determined through single-locus statistical tests, such as odds–ratio, *χ* ^2^, or Fisher's exact text (so-called weighted risk scores). While such standard models have provided relatively good predictive accuracies, as assessed using simulation studies or hypothetical effect size distributions [29], we argue here that it makes sense to use machine learning both for selecting the subsets of the most predictive genetic features as well as training the final prediction model using regularized learning approaches [5], [30]. The recent work of Chatterjee et al., where they estimated the effect size distributions for various quantitative and disease traits, highlighted the benefits gained from more holistic models that make use of the whole spectrum of genetic variation toward improving the predictive power of the genetic risk prediction models [31]. By design, the performance of any prediction model will depend on the sample size of the training set, as well as heritability of the disease trait, its underlying genetic architecture, and whether there is additional information available such as family history [29]–[33].

## Representative Examples of Supervised Predictive Modeling Studies

Predictive modeling can be treated either as a classification problem (e.g., disease prediction in a case-control setting) or as a regression formulation (e.g., prediction of height in a general population cohort). Regardless of the problem formulation, however, the critical issue is how to guarantee that the model estimated in the training sample enables generalization power on new sets of individuals using appropriate learning models and regularization approaches. Another important issue is how to evaluate and quantify the predictive performance of these models using procedures such as cross validation (CV) and statistics such as the area under the curve (AUC) or coefficient of determination (*R* ^2^) (Text S1). These factors are next highlighted using representative examples from the recent literature [1]–[4], [34], [35], where various machine learning models have been implemented to gain insights and prediction capability beyond that obtained using standard statistical analyses of single nucleotide polymorphism (SNP) data.

In one of the first machine learning applications, Wei et al. showed that support vector machines (SVM) and *L*_2_-regularized (ridge) logistic regression enabled construction of a highly predictive risk model for type 1 diabetes (T1D) using less than 500 variants that passed a relatively stringent prefiltering threshold (*p*<10^−5^) on a case-control GWA dataset [1]. In contrast, relying merely on a collection of known T1D susceptibility loci led to poor performance in the predictive setting. More specifically, when the predictive accuracy was evaluated in terms of within-study 5-fold CV, they obtained extremely good prediction power (AUC close to 0.9). However, it is known that simple CV may lead to over-optimistic results due to information leakage between the two stages of the feature selection process [5]. Indeed, when the predictive models were evaluated using totally independent validation cohort, the between-study performance dropped drastically (AUC 0.84 for SVM) [1], highlighting the importance of independent samples in the model validation.

Recently, Wei et al. made use of larger sample sizes (>10,000 individuals), using variant data from 15 European countries for risk prediction of Crohn's disease (CD) and ulcerative colitis (UC) [4]. They applied a custom Immunochip that provides a more comprehensive catalog of both common variants and certain rare variants missed in the first generation of GWA studies. Using a relatively liberal threshold (*p*<10^−4^), they preselected around 10,000 variants and applied regularized logistic regression with *L*_1_ penalty for sparse genetic risk modeling. In an independent validation set from the meta-analysis cohort, the predictive models achieved the best prediction performance reported for CD and UC (AUCs of 0.86 and 0.83, respectively) so far. In contrast, the simple odds–ratio-weighted genetic risk model showed relatively poor results (AUC of 0.730 and 0.685, respectively). The study also confirmed the projections from previous works [31]–[33], suggesting that predictive accuracy is highly dependent on the sample sizes and the spectrum of variants included in the model, in addition to the heritability of the disease trait.

The final example comes from the regression formulation. With the aim to explain a part of the missing heritability of height, Yang et al. [34] went beyond the two-stage approach and fit a simple linear regression model to all directly genotyped 294,831 variants that passed their quality control. Using such a whole genome prediction (WGP) approach, without any variant selection, the authors were able to explain 45% of the phenotypic variation in height in a cohort of approximately 4,000 European descents. Similarly high *R* ^2^ values were also confirmed in another study [35] where the WGP approach was trained in an European cohort; however, *R* ^2^ values dropped dramatically when the fitted model was applied to an independent validation dataset using 10-fold CV (*R* ^2^ ranging around 0.2, depending on the number of variants and whether familial information was used) [35]. These studies highlight the risk of overfitting to the training sample when no feature selection or model regularization is used in the model construction.

## Prediction Performance Using Examples of Model Regularization

**Figure 1:**
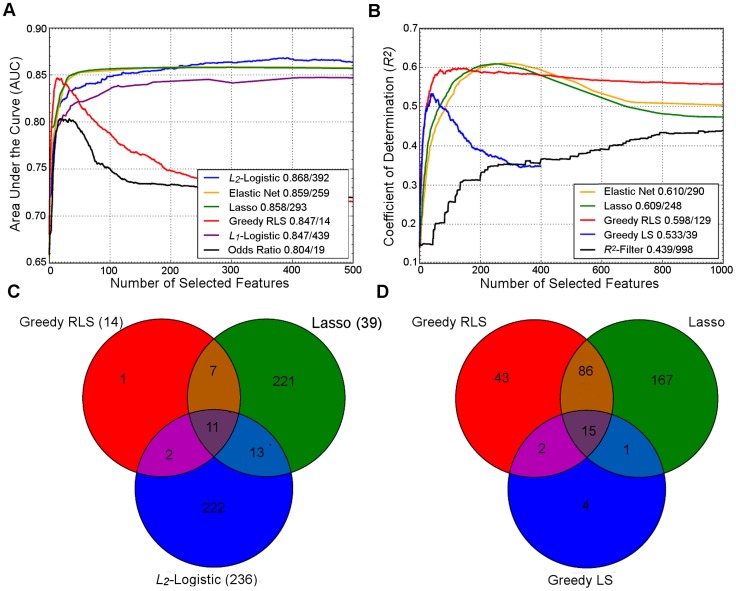
Performance of regularized machine learning models. Upper panel: Behavior of the learning approaches in terms of their predictive accuracy (*y*-axis) as a function of the number of selected variants (*x*-axis). Differences can be attributed to the genotypic and phenotypic heterogeneity as well as genotyping density and quality. (A) The area under the receiver operating characteristic curve (AUC) for the prediction of Type 1 diabetes (T1D) cases in SNP data from WTCCC [118], representing ca. one million genetic features and ca. 5,000 individuals in a case-control setup. (B) Coefficient of determination (*R* ^2^) for the prediction of a continuous trait (Tunicamycin) in SNP data from a cross between two yeast strains (Y2C) [44], representing ca. 12,000 variants and ca. 1,000 segregants in a controlled laboratory setup. The peak prediction accuracy/number of most predictive variants are listed in the legend. The model validation was implemented using nested 3-fold cross-validation (CV) [5]. Prior to any analysis being done, the data was split into three folds. On each outer round of CV, two of the folds were combined forming a training set, and the remaining one was used as an independent test set. On each round, all feature and parameter selection was done using a further internal 3-fold CV on the training set, and the predictive performance of the learned models was evaluated on the independent test set. The final performance estimates were calculated as the average over these three iterations of the experiment. In learning approaches where internal CV was not needed to select model parameters (e.g., log odds), this is equivalent to a standard 3-fold CV. T1D data: the *L*_2_-regularized (ridge) regression was based on selecting the top 500 variants according to the *χ* ^2^ filter. For wrappers, we used our greedy *L*_2_-regularized least squares (RLS) implementation [30], while the embedded methods, Lasso, Elastic Net and *L*_1_-logistic regression, were implemented through the Scikit-Learn [119], interpolated across various regularization parameters up to the maximal number of variants (500 or 1,000). As a baseline model, we implemented a log odds-ratio weighted sum of the minor allele dosage in the 500 selected variants within each individual [25]. Y2C: the filter method was based on the top 1,000 variants selected according to *R* ^2^, followed by *L*_2_-regularization within greedy RLS using nested CV. As a baseline model, we implemented a greedy version of least squares (LS), which is similar to the stepwise forward regression used in the original work [44]; the greedy LS differs from the greedy RLS in terms that it implements regularization through optimization of *L*_0_ norm instead of *L*_2_. It was noted that the greedy LS method drops around the point where the number of selected variants exceeds the number training examples (here, 400). Lower panel: Overlap in the genetic features selected by the different approaches. (C) The numbers of selected variants within the major histocompatibility complex (MHC) are shown in parentheses for the T1D data. (D) The overlap among then maximally predictive variants in the Y2C data. Note: these results should be considered merely as illustrative examples. Differing results may be obtained when other prediction models are implemented in other genetic datasets or other prediction applications.

To illustrate the similarities and differences in their behavior, we ran a number of common regularization approaches on two example datasets (Figure 1). In both datasets, the two embedded methods, Lasso and Elastic Net, showed strikingly similar prediction behavior, but needed a larger number of variants for their peak performance, compared to the greedy regularized least-squares (RLS) wrapper, which peaked much earlier but resulted in lower prediction accuracy. As was expected, the top performance of the *L*_2_-regularized logistic (ridge) regression required a very large number of features, while showing reduced accuracy at a lower number of variants. Surprisingly, the popular *L*_1_-penalized logistic model showed slightly suboptimal performance; although its peak performance was similar to that of greedy RLS, it required a much larger number of variants in these datasets. We note that the relative behavior of these methods may well change in other genetic datasets and applications. In line with the previous results in CD and UC cases [4], the simple log odds-weighted risk model also showed poor results in the T1D case. While for some other traits such accuracies would be considered excellent, the high heritability and dependence on the human leukocyte antigen (HLA) region often leads to higher predictive performance for T1D [1]. However, these accuracies are better than expected for a sample of this size if the standard, nonmachine learning, multilocus genetic models were utilized in the risk prediction [28].

**Figure 2:**
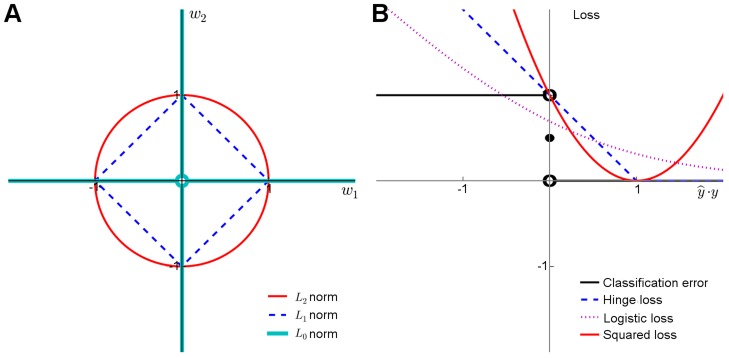
Penalty terms and loss functions. (A) Penalty terms: *L*_0_-norm imposes the most explicit constraint on the model complexity as it effectively counts the number of nonzero entries in the model parameter vector. While it is possible to train prediction models with *L*_0_-penalty using, e.g., greedy or other types of discrete optimization methods, the problem becomes mathematically challenging due to the nonconvexity of the constraint, especially when other than the squared loss function is used. The convexity of the *L*_1_ and *L*_2_ norms makes them easier for the optimization. While the *L*_2_ norm has good regularization properties, it must be used together with either *L*_0_ or *L*_1_ norms to perform feature selection. (B) Loss functions: The plain classification error is difficult to minimize due to its nonconvex and discontinuous nature, and therefore one often resorts to its better behaving surrogates, including the hinge loss used with SVMs, the cross-entropy used with logistic regression, or the squared error used with regularized least-squares classification and regression. These surrogates in turn differ both in their quality of approximating the classification error and in terms of the optimization machinery they can be minimized with (Text S1).

The relatively small overlap in the selected features highlights an interesting point that the models tend to select different panels of variants while achieving rather similar prediction performance (Figure 1C, D), suggesting that the selected variants may provide complementary views of the genetic mechanisms behind the phenotypes. In the T1D case, for instance, most of the variants selected by the *L*_2_-logistic and greedy RLS were from the major histocompatibility complex (MHC) region (95% and 67%, respectively), in line with the previous studies [1], [4], whereas Lasso also selected novel variants mostly outside the MHC region (15%), which may provide complementary information for the risk assessment. This difference is likely due to its embedded nature; Lasso selects variants simultaneously, rather than one at a time, which often requires further optimization in applications where the size of the variant panel is limited. As expected, the univariate filters tend to select larger numbers of correlated features, since they cannot consider interactions with already selected variants. At the other extreme, greedy RLS selects relatively uncorrelated variants while the embedded methods lie in between. These example cases suggest that there is no golden rule for feature selection, but that the model should be selected based on the characteristics of the data and goals of the genetic application (e.g., whether small number of variants is preferred over the overall predictive accuracy).

## Perspective: Current Challenges and Emerging Developments

While rare variants have been proposed as one explanation for the missing heritability [36], [37], there has been a divergence of opinion over whether rare variants of large effect or common variants of small effect are contributing most to the phenotypic variability [38]. It has been suggested that incorporating low-frequency or rare variants will make the disease risk prediction increasingly more accurate [4], [28], [29], [31]. However, recent reports have shown only incremental impact of rare variants on disease susceptibility and prediction of complex diseases, as evaluated at the population level using either simulated data [39] or by sequencing of known risk variants for autoimmune disease traits [40]. We believe that a more systematic investigation of the variants across portions of the allelic spectrum will likely contribute to explaining more of the missing heritability. While the presented machine learning algorithms easily scale to a GWA level, the emerging sequencing data, either from genotype imputation or whole-exome and genome profiling, are posing new technical challenges, where parallelization and cloud technologies for distributed memory and high-performance computing will become increasingly important. Placing the focus on individual-level predictions should help also with the low-frequency variants shared only by a small portion of the individuals. For instance, selection of the most robust variants was shown to improve various prediction models, especially when the variants are poorly tagged or have low minor allele frequency (MAF) [41]. Since most rare variants are highly population-specific, it may be necessary to borrow prior biological information from shared regulatory regions, genes, or pathways, similar to the recent collapsing methods for rare association analyses [42]. However, improved model regularization options that allow more flexibility and sparsity in the selected panels of variants across various subgroups of individuals will likely be needed to deal with the rare variants and to account for population stratification. Regularization methods based on sparse group Lasso, for instance, can be extended to rare variants and pathway-driven variant selection [22], [43].

It has been argued that, even with increasingly large-scale and dense genomic data, genetic prediction alone may still not reach the accuracy regarded as clinically informative for the population at large [18]. High-quality and controlled genetic data from model organisms will likely give the first estimates on how much sequencing data can really add to the predictive accuracy of complex phenotypes [44], [45]. Lessons from model organisms have already shown that additional information originating from environmental and stochastic factors, as well as from phenotypic robustness and transgenerational effects, will be necessary for accurate predictions at an individual level [46]–[48]. In particular, gene expression should prove especially useful, since such intermediate phenotype captures both genetic and nongenetic contributions to phenotypic variation [49]. For instance, epigenetic gene expression variability of genetic interaction partners plays an important role in explaining complex regulatory relationships, characterized using concepts such as “epigenetic epistasis” [50] or “eQTL epistasis” [51]. Although modeling of gene expression variability poses some technical challenges, similar to those already encountered when modeling GWA datasets [52], [53], incorporating such continuous features into the disease prediction models should be relatively straightforward. Adding the nongenetic information will likely be instrumental when going toward less heritable diseases, such as some cancer subtypes, which traditionally have been challenging to predict using standard GWA approaches [29], [32], [33], [54]–[56]. Finally, including family medical history and other clinical data from electronic health records should improve the personal risk assessment models, as well as provide guidance on lifestyle changes for those currently healthy individuals that have increased genetic risk for the disease susceptibility [57], [58].

An interesting question under debate is how many genetic features should be incorporated into the prediction models [3], [28], [31], [59], [60]. Although the WGP methods have been successfully applied in animal and plant breeding applications [61], these are not suitable for applications in which the number of genetic markers is constrained. In embedded models, the number of features to be selected is often dependent on the regularization parameter. However, in the current Lasso and Elastic Net implementations, the user cannot explicitly specify the number of variants to be included in the final model, but the selection of final predictors often requires further grid searches or other tuning options. Such lack of direct control over the size of the variant panel may be an important practical consideration in medical applications, where the size of the variant panel is often associated with an additional cost, for instance, in disease screening applications, or when the goal is to select a few of the variants for follow-up experimentation, for instance, using functional assays. Greedy feature selection offers full control to the user and often leads to smaller panels of predictive, uncorrelated variants, which may be beneficial when the size of clinical assay is limited. However, the trade-off is a slight drop in the overall predictive accuracy (Figure 1), indicating that more in-depth and effective wrapper selection strategies need to be implemented. There are also other strategies to reduce the dimensionality of genetic feature spaces using data transformations, such as principal components analysis (PCA), multidimensional scaling (MDS), partial least squares (PLS), or discrete wavelet transformation (DWT), which may in some cases lead to improved predictive accuracy [62]. However, rather than selecting combinations of transformed features, feature selection on the original variant space offers directly actionable modeling outcomes, such as a selected set of predictive genetic loci for follow-up applications and experimentation.

We envision a number of future directions for improvements in disease risk prediction. One exciting development involves modeling of cross-phenotype interactions (pleiotropy). Many genetic variants are associated with multiple disease phenotypes, particularly across autoimmune diseases, cancers, and neuropsychiatric disorders [63]. Statistical approaches have been suggested for making use of the complementary information from multiple phenotypes to gain power to detect small effects that would have been missed if tested individually [64]–[65]. Bayesian learning approaches seem particularly fitting for multivariate modeling of pleiotropic associations, especially for the lower-frequency variants where shared genetic features across individuals for any single phenotype become increasingly rare [66]–[71]. We expect that regularized machine learning models will also prove useful when translating the subtle multivariant–multiphenotype relationships into genetic risk prediction models. Modeling studies in yeast have already shown that multiple phenotypic measurements enable mapping of genetic interaction networks with distinct biological processes across pathways [72]. Networks of genetic and/or physical interactions may therefore serve as useful prior information for the prediction models to move from variant-level features towards pathway-level features [5], [73]–[75]. Using such functional relationships to assemble or collapse higher-level predictive features might better account for the interindividual genetic variation at the lower end of variant frequency. For instance, predictive subnetwork modules could enable more robust personalized medicine strategies by allowing that individuals with the same disease phenotype may show interindividual genetic heterogeneity in the sense that their disease predisposing variants may lie in distinct loci within the shared pathways. Such advances will rely on the next generation of machine learning models that can effectively deal with the complexity arising from massive number of interactions between rare and common genetic and nongenetic factors [76]–[78].

## Conclusions

The current evidence contradicts the idea of a universally optimal model across datasets and prediction applications; rather, the model should be selected based on whether one is trying to achieve a maximally predictive model without restricting the number or type of variants, or whether the goal is to build a sufficiently predictive model with a limited number of genetic and nongenetic features. This highlights the importance of feature selection as a key component in the construction of prediction models, whether it is done explicitly in the optimization process (e.g., wrappers) or implicitly through the model regularization (embedded models). One common finding is that those variants not meeting the stringent genome-wide significance levels may also contribute to the predictive signals when combined in the multilocus prediction modes [2], [4], [24], [25], [27], [28], [31], [33]. Another consensus point is that regularized models often outperform their unregularized counterparts [24], which was also supported by our example results (Figure 1).

Regardless of the model used, however, careful evaluation of its generalizability is critical for prediction applications. We encourage using systematic and unbiased procedures, such as nested CV, for the selection of genetic variables and other model parameters and for the evaluation of the generalization performance of the model. The final model construction and feature selection should be performed on the complete set of samples using standard CV options. However, the eventual predictive power must be assessed by implementing the final model on a sufficiently large, representative, and independent test set in order to avoid reporting over-optimistic prediction results. The model evaluation also depends on the application case; for instance, if the aim is to carry out disease screening in Finland, then a relatively large Finnish population sample should be used both in the model construction and validation.

Genetic risk prediction through supervised machine learning models goes beyond the single-locus association testing with the complex disease phenotypes. The main objective of regularized learning approaches is to find the most predictive combinations of variants, the functional roles of which must to be validated using follow-up experimentation. However, it is likely that predictive power is linked to the underlying biological mechanisms and even causality, but whether this comes through the selected variants and their interactions, or via synthetic associations or other nondirect relationships needs to be evaluated mechanistically. Genotype–phenotype modeling is a highly challenging problem, but we believe that through appropriate implementation and application of the supervised machine learning methods, such as those presented here, increasingly predictive relationships and biological understanding will be extracted from the current and emerging genetic and phenotypic datasets.

## Supporting Information

Text S1 Implementation details for a range of regularized machine learning models.
(PDF)
