# Supplementary material for: Regularized Machine Learning in the Genetic Prediction of Complex Traits
Source: PLoS Genet. 2014 Nov 13;10(11):e1004754. doi: 10.1371/journal.pgen.1004754 (PMC4230844; doi:10.1371/journal.pgen.1004754)
Supplement: Text S1 — Implementation details for a range of regularized machine learning models. (PDF) [file pgen.1004754.s001.pdf]

Implementation details of regularized machine learning models

This work aims to infer a model for making predictions for the disease class status through the use of supervised machine learning and feature selection techniques [1–4]. We assume that we are given a training set of m examples, each consisting of an n-dimensional feature vector, representing the SNP data and an associated outcome label. The aim of supervised learning is to infer a model from the training data that would allow predicting the labels for new data, for which only the features data is provided. Formally, we define the training set $\mathcal{T} = \{(\boldsymbol{X}_{1,:}, \boldsymbol{y}_1), \ldots, (\boldsymbol{X}_{m,:}, \boldsymbol{y}_m)\}$, where $\boldsymbol{y}_i \in \mathbb{R}^m$. By $\boldsymbol{X} \in \mathbb{R}^{m \times n}$ we denote a data matrix containing the feature vectors as rows and by $\boldsymbol{y}$ we denote the m-dimensional vector containing all the training set labels. By $\boldsymbol{X}_{i,:}$ we denote the i:th row, and by $\boldsymbol{X}_{:,i}$ the i:th column of $\boldsymbol{X}$. Our aim is to learn a prediction function $h : \mathbb{R}^n \rightarrow \boldsymbol{y}$ such that for new input-output pairs $(\mathbf{x}, y)$ it holds true that $h(\mathbf{x}) \approx y$.

Based on the choice of y we can recover a variety of different supervised learning settings. In binary classification, typically encoded as $y \in [-1, 1]$, we have two possible classes called the positive and the negative class. This is the standard way for modeling case-control studies where individuals belong to one of two classes based on whether they have a disease or not. In settings where there is a natural ordering over the classes, for example when the classes represent different stages of a disease in progression, the problem is known as ordinal regression. Finally, when $y \in \mathbb{R}$, we are presented with the problem of regression, where the aim is to learn to predict a real-valued variable, such as individual's blood pressure or weight.

In practice the model h is often implemented using one or several real-valued prediction functions $f : \mathbb{R}^n \rightarrow \mathbb{R}$. This is natural for regression, while binary classification may be implemented as a decision rule, for example it can defined by a transformation $h : \mathbb{R} \rightarrow \{-1, 1\}$ such that

$$h(q) = \begin{cases} 1, & \text{if } q > t \\ -1, & \text{if } q \leq t \end{cases} .$$

Here, one may as a default set $t = 0$ when aiming to minimize the misclassification cost, or choose some other threshold value when the misclassification costs between the two classes are known to be asymmetric.

Filter Methods for Genetic Feature Selection

The simplest form of feature selection that is commonly applied to GWAS is known as filter methods. Filters make use of the intrinsic properties of an individual's genetic variants to determine the bearing of the feature on the phenotype [5]. When features are selected by filter methods, the set of genetic variants are evaluated individually with a test statistic to determine their predictability for a particular phenotype. Let $\mathcal{S}$ denote the set of selected features, and $\mathcal{P}(\boldsymbol{X}_{:,i}, \boldsymbol{y})$ the value of some univariate statistic, that computes the error obtained when using the ith feature, whose value for all training examples is contained in $\boldsymbol{X}_{:,i}$, for predicting the corresponding labels contained in $\boldsymbol{y}$. It can be assumed that a lower value for the statistic means better predictive power, as we can usually define simple transformations for statistics that do not behave this way (i.e. use 1-accuracy as an error measure instead of using accuracy). A number of studies have shown that filters were able to provide predictive results for their respective datasets [3, 6]. Some studies have coupled these filters with more advanced methods such as wrappers [2, 7]. These studies collectively argue that through the intelligent use of these algorithms researchers can help to explain a larger portion of the heritability of complex diseases and help to find more causal variants for these phenotypes.

Algorithm 1 presents the general pseudocode for the filter method. Those SNPs for which the value of the statistic is below a certain threshold are selected. Further, one may restrict the method to selecting only the top k variants, in which case the features need to be ranked by sorting the computed values. Running algorithm 1 can be implemented highly efficiently, as they require only a single pass through the data. In practice, standard GWAS analysis software such as PLINK [8] are capable of calculating single-locus statistical associations for entire GWAS in only a matter of minutes.

Algorithm 1 Filter-based feature selection

```
S ← ∅                                   ▷ The set of selected features
for i ∈ {1, ..., n} do
    ε_i ← P(X_{:,i}, y)                 ▷ Calculate the value of the statistic P
    if ε_i < ε_t then                   ▷ Select the feature if the value is below threshold ε_t
        S ← S ∪ {i}
return S
```

There are a number of commonly used filters and which method to use is a subject of determination as to what provides the most meaningful results for a particular study. In case-control studies, a common analysis is to test the hypothesis of no-association between the SNP's values in cases and controls, represented in a 2×3 matrix (see Table 1). This matrix contains the counts of the three genotypes in the different subgroups. A less computationally intensive method is to treat the contingency table as 2×2 in which the entries represent the count of the total number of the allele possibilities in cases and controls (see Table 2).

	**AA**	**Aa**	**aa**	**Total**
**Cases**	z_1	z_2	z_3	R_1
**Controls**	z_4	z_5	z_6	R_2
**Total**	C_0	C_1	C_2	N

Table 1: Example of 2x3 genotypic table

	**A**	**a**	**Total**
**Cases**	$2z_1 + z_2$	$2z_3 + z_2$	$2R_1$
**Controls**	$2z_4 + z_5$	$2z_6 + z_5$	$2R_2$
**Total**	$2C_0 + C1$	$C_1 + 2C_2$	$2N$

Table 2: Example of 2x2 observed genotypic table

	**A**	**a**
**Cases**	$R_1(2C_0 + C_1)/N$	$R_1(C_1 + 2C_2)/N$
**Controls**	$R_2(2C_0 + C_1)/N$	$R_2(C_1 + 2C_2)/N$

Table 3: Example of 2x2 expected genotypic table

Given that the tables contain r rows and c columns, with R_i and C_j denoting the sums of the entries of row i and column j, respectively, the p-value for Fisher's Exact Test can be calculated with an application of the following equation [9].

$$p = \frac{\prod_{b=1}^{r} R_b! \prod_{d=1}^{c} C_d!}{N! \prod_{i=1}^{c \times r} z_i}. \tag{1}$$

Similar to Fisher's Exact test, one can also use the χ^2 test statistic to calculate the association between individual SNPs and the correct class labels. Based on the observed genotypes in Table 2 and the expected ones in Table 3, the test statistic can be computed with $(r-1)(c-1)$ degrees of freedom:

$$\chi^2 = \sum_{i=1}^{r} \sum_{j=1}^{c} \frac{(Obs_{ij} - Exp_{ij})^2}{Exp_{ij}}$$

Another commonly used metric is known as the odds ratio (OR) which measures the association of a variable on the class labels. This measure indicates how likely a given class label will be based on an

observed genotype compared to the likelihood of that same label without the appearance of the particular genotype [10]. Using Table 2 we can define the allele count based OR:

$$OR = \frac{(2z_1 + z_2)(2z_6 + z_5)}{(2z_3 + z_2)(2z_4 + z_5)}$$

If the OR = 1, it can be assumed that there is no association between the genotype and the disease. A value of OR > 1 represents that allele **A** increases the risk of the disease and an OR < 1 means that the occurrence of the disease is less likely.

Coordinate Descent Optimization for Feature Selection

With wrapper and embedded feature selection, we refer to methods for which the selection process is optimized for a particular learning algorithm. In traditional wrapper methods, feature selection is implemented as a meta-algorithm that performs feature selection as a search over the power set of features, and the learning algorithm is used as a black-box subroutine that evaluates the quality of different feature sets. Embedded methods on the other hand incorporate feature selection within a particular machine learning algorithm, for example by changing the objective function optimized so that it favors sparsity in addition to prediction performance. Wrapper methods can be considered as more general, whereas embedded methods are typically more efficient as they allow algorithm specific optimizations in implementing the methods. It is not always possible to draw clear distinctions between these two classes of approaches, as a specific optimized realizations of the general wrapper framework may in many cases be considered as embedded algorithms, as is the case for example the greedy RLS method considered later in this section.

We next present an optimization framework for wrapper and embedded methods, under which various types of feature selection methods can be conveniently considered. The linear models considered in the framework can be written as

$$f(\boldsymbol{x}) = \boldsymbol{x}^{\mathrm{T}}\boldsymbol{w} + b \tag{2}$$

where $\boldsymbol{w} = (w_1, \ldots, w_n)^{\mathrm{T}}$ is a vector of model parameters, b is the bias and $\mathbf{x} = (x_1, \ldots, x_n)^{\mathrm{T}}$ is a vector containing the feature values of a datum. Typically the bias term b is implemented by appending to each feature vector $\boldsymbol{x}$ a constant valued feature $\boldsymbol{x}_0 = 1$, so that we may define $\boldsymbol{w}_0 = b$, as this simplifies the notation. When dealing with this data representation, we assume that the feature selection algorithms always automatically select the feature corresponding to the bias term. Thus, the model (2) will be subsequently written without the bias term.

The training algorithms for learning the above considered types of linear models can be expressed as a following optimization problem:

$$\begin{aligned} &\underset{\boldsymbol{w}\in\mathbb{R}^n}{\operatorname{argmin}} \textstyle\sum_{i=1}^{m} L\left(f(\boldsymbol{X}_{i,:}), \boldsymbol{y}_i\right) \\ &\text{subject to } C(\boldsymbol{w}) , \end{aligned} \tag{3}$$

where L is a loss function indicating how the prediction obtained for the ith training example fits to its label, and C is a constraint function. One of the most well-known and most widely used constraint functions is the so-called quadratic (or ridge) regularizer

$$C(\boldsymbol{w}) \equiv \|\boldsymbol{w}\|_2^2 < r, \tag{4}$$

where $r \in \mathbb{R}^+$. Constraining the norms of the models is traditionally used for finding a balance between fitting the model to the training data and the complexity of the model. However, this constraint alone tends to favor models that depend on all features.

One of the simplest sparsity enforcing constraints is the one setting a hard limit on the number of features the model can depend on, that is, on the number of nonzero entries in the model vector $\boldsymbol{w}$, known in the

literature as the zero norm $\|\boldsymbol{w}\|_0 = |\{i \mid w_i \neq 0\}|$ of the model vector. This can be formally expressed as follows:

$$C(\boldsymbol{w}) \equiv \|\boldsymbol{w}\|_0 \leq k, \tag{5}$$

where $k \in \mathbb{N}$ is a user given limit which the number of features must not exceed. The discrete and non-convex nature of the constraint (5) makes its direct optimization challenging, and one must resort to combinatorial optimization techniques, such as discrete searches over the space of all feature subsets. In the literature, the methods are often referred to as wrapper-based feature selection methods, which originate from the idea of retraining a model from scratch for each step of the search process. Namely, the search algorithm is "wrapped" around a base training algorithm that outputs a new model for each tested subset of features. This can be very slow in practice due to the size of the search space growing exponentially in the number of features and due to the slow training speed of the base learning algorithms. As we will show below in more detail, the large search space can be countered by designing smart search heuristics and the training speed can be accelerated by taking advantage of the models trained during the previous iterations of the search algorithm. An extra benefit of the direct search of feature subsets is that, instead of optimizing the training error, one can also optimize more sophisticated objectives, such as the leave-one-out cross-validation error for which some learning algorithms have easy to optimize closed-form solutions.

One of the oldest computationally efficient algorithms for directly optimizing the least-squares loss with the discrete constraint (5) are the so-called greedy least squares (GLS) methods, also known as orthogonal matching pursuit [11]. Another typical example of a direct optimization approach is the greedy RLS algorithm proposed by us [12]. It uses both (4) and (5) simultaneously to constrain the space of the models, leave-one-out cross-validation as a search heuristic and combinatorial search as an optimization method.

Algorithm 2 Greedy coordinate descent

$\mathcal{S} \leftarrow \emptyset$
while $|\mathcal{S}| < k$ **do**
  $b, r \leftarrow \text{argmin}_{b \in \{1,\ldots,n\} \setminus \mathcal{S}, r \in \mathbb{R}} J(\boldsymbol{w} + r\boldsymbol{e}_b)$
  $\mathcal{S} \leftarrow \mathcal{S} \cup \{b\}$
  $\boldsymbol{w} \leftarrow \boldsymbol{w} + r\boldsymbol{e}_b$
return $\mathcal{S}$

This type of approaches can be conveniently considered under the framework of coordinate descent methods. Coordinate descent (see e.g. [13]) fixes all entries of the vector except one and updates its value so that the value of the objective function will be reduced. A greedy coordinate descent is illustrated in Algorithm 2. The algorithm starts from an initial set of features that can be an empty set as in greedy forward selection and other similar approaches. During each iteration the algorithm prepares a set of candidate feature sets that usually differ only slightly from the current set of selected features. The candidates cover all subsets that have one extra feature in addition to the features already selected. Next, the algorithm selects from the candidates the one that is optimal with respect to the function to be optimized and subsequently updates the model according to the new set of selected features. The algorithm stops when a predefined number of features k have been selected, or based on other varying criteria, such as the identification of an optima.

Instead of directly optimizing the hard constraint of type (5), an alternative approach is to approximate it with an easier to optimize proxy function, such as the so-called 1-norm of the model vector

$$C(\boldsymbol{w}) \equiv \|\boldsymbol{w}\|_1 \leq r. \tag{6}$$

Unlike the 2-norm based constraint (4) this tends to favor sparse models depending only on a subset of the original features, and unlike (5), this is a convex and continuous constraint. Combined with a convex loss function, the corresponding optimization problem is considerably easier to solve than those with discrete non-convex constraints due to the objective function having a global optimum easily searchable with the powerful family of convex optimization methods.

Algorithm 3 Cyclic coordinate descent

while not converged **do**
  for $j = 1, \ldots, n$ **do**
    $w_j \leftarrow w_j + \text{argmin}_{r \in \mathbb{R}} J(\boldsymbol{w} + r\boldsymbol{e}_j)$

The methods resorting to the convex approximation constraint (6), are usually called the embedded methods, since the feature selection mechanism can be considered to be built into the training algorithm itself. While there is a long history of various convex optimization methods being applied for training the embedded methods, currently the most popular ones are coordinate descent algorithms due to their simplicity and computational efficiency.

In this case, cyclic coordinate descent may be applied for minimizing the objective function (see Figure 1). The method repeats coordinate descent steps for each coefficient in the model vector at a time in a cyclic fashion, until the solution has converged or if a pre-defined maximum number of passes through the whole data has been performed. The idea is illustrated in Algorithm 3, where J denotes the constrained objective function and $\boldsymbol{e}_j$ is the jth standard basis vector of $\mathbb{R}^n$ (e.g. the jth element of $\boldsymbol{e}_j$ is 1 while the other entries are zero).

The most well-known algorithm involving the constraint (6) is known as Lasso or basis pursuit in the fields of machine learning and signal processing, respectively. Elastic Net is a variation of Lasso that simultaneously uses both (4) and (6). Lasso and Elastic Net are both least-squares regression methods but ℓ_1-regularization has also been employed together with other loss functions such as the logistic loss for binary classification. There has recently been growing sectors of research that have made use of embedded methods, primarily Lasso and similar ℓ_1-based methods, for the development of predictive models [4, 14–18].

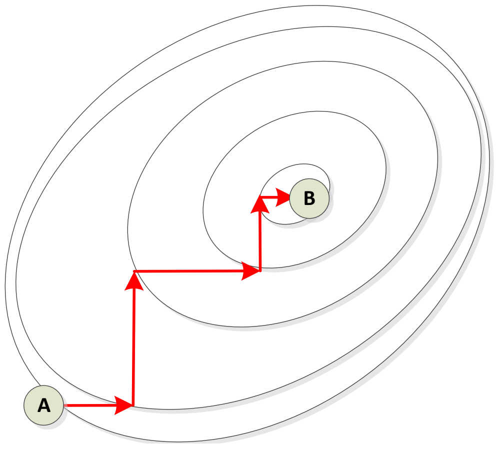

Figure 1: Example of coordinate descent starting from A and searching for the minimum B of a convex function.

Learning Algorithms

Let us denote $p = f(\mathbf{x}) = \langle \mathbf{x}, \boldsymbol{w} \rangle$, thus

$$\frac{\partial p}{\partial w_j} = x_j.$$

Then by chain rule for any loss function

$$\frac{\partial L(p, y)}{\partial w_j} = \frac{\partial L(p, y)}{\partial p} \frac{\partial p}{\partial w_j} = x_j \frac{\partial L(p, y)}{\partial p}.$$

From these we can define the derivatives needed for finding the coordinate descent step directions for a number of loss functions (see table 4)[1]. Based on these and the various regularizers listed in Table 5 we can construct a number of well-known machine learning algorithms, as seen in Table 6. Note that, while the zero-norm is not differentiable, the corresponding constraint is still satisfied with the greedy coordinate descent based training methods. Different algorithms vary on their respective computational complexities (see table 7).

Name	$L(p, y)$	$\frac{\partial L(p,y)}{\partial p}$
Squared	$(p-y)^2$	$2(p-y)$
Logistic	$\log(1+e^{-yp})$	$\frac{-y}{1+e^{yp}}$
Hinge	$\max(0, 1-yp)$	0 if $y \geq 1$, $-y$ else

Table 4: Common loss functions

Name	$\Omega(\boldsymbol{w})$	$\frac{\partial \Omega(\boldsymbol{w})}{\partial w_i}$
ℓ_0	$\|\boldsymbol{w}\|_0$	–
ℓ_1	$\|\boldsymbol{w}\|_1$	$\mathrm{sign}(w_j)$
ℓ_2	$\|\boldsymbol{w}\|_2^2$	$2w_j$

Table 5: Common regularizers

Method	S	L	H	ℓ_1	ℓ_2	ℓ_0	R/C	Ref.
Lasso	•			•			R	[19]
Elastic Net	•			•	•		R	[20]
ℓ_1 Logistic		•		•			C	[21]
ℓ_2 Logistic		•			•		C	[22]
ℓ_1 SVM			•	•			C	[23]
SVM			•		•		C	[24]
OLS	•						R	[25]
Greedy RLS	•				•	•	R	[12]
Ridge Reg	•				•		R	[26]
GLS	•					•	R	[11]

Table 6: Construction of various methods based on different loss functions and regularizers. *S*, *L* and *H* stand for squared loss, logistic loss and hinge loss, respectively. R/C denotes whether the method is a (R)egression or a (C)lassification method only, all the regression methods can also be used for classification. OLS stands for ordinary least squares and *Ridge Reg* for ridge regression. *GLS* represents greedy least squares. These methods may also be known by other names in the literature, for example ridge regression is also known as regularized least squares.

[1]To be exact, the hinge loss and ℓ_1 norm are not differentiable everywhere, for these we provide subderivatives.

Method	Complexity
Greedy RLS	$O(kmn)$
Filter	$O(mn)$
Cyclic Coordinate Descent	$O(mnD)$
Greedy Coordinate Descent	$O(kmn)$

Table 7: Computational complexities of various feature selection methodologies. Here D represents the number of iterations necessary for the algorithm to cycle through until convergence. This is a data dependent variable and will vary depending on the study examined.

Examples in genetic prediction of complex traits

Next, we consider representative examples of implementations of the previously considered optimization framework, such that allow feature selection and can scale to entire GWAS without the need for pre-filtering. Greedy least squares (GLS) [11], minimizes the squared loss with a zero-norm constraint, e.g. the number of nonzero features is restricted. A straightforward approach for training GLS is to use the greedy coordinate descent (see Algorithm 2), which greedily selects one feature at a time and updates the model accordingly, until the number of features determined by the constraint is selected. The method is very simple to implement and the greedy search steps can be accelerated by caching the results from previous iterations. Accordingly, the computational complexity is only linear with respect to the number of features, data and the constraint (Table 7). The squared loss for m data will become zero at the latest after m linearly independent features has been selected, and hence GLS cannot be used to select more than that.

Greedy RLS [12] is similar to GLS except that it uses a combination of zero- and two-norm constraints and the it is trained with greedy coordinate descent that optimizes the leave-one-out cross-validation error rather than a traditional type of objective function. That is, the method yields identical results to running a traditional greedy forward feature selection wrapper on quadratically regularized least-squares (RLS) (e.g. ridge regression), but does so with computational shortcuts that are similar to those used in embedded methods. Formally, the selection heuristic $\mathcal{H}$ is the leave-one-out cross-validation error measured on RLS trained with features $\mathcal{S} \cup \{j\}$. Formally, it can be expressed as

$$\mathcal{H}(\mathcal{S} \cup \{j\}) = \sum_{i=1}^{m} \left(\boldsymbol{X}_{i,:}\boldsymbol{w}^{(i)} - \boldsymbol{y}_i \right)^2 , \tag{7}$$

where $\boldsymbol{w}^{(i)}$ is the RLS model trained with the whole training dataset except the ith datum and using the features indexed by $\mathcal{S} \cup \{j\}$, that is, the minimizer of

$$\sum_{h \neq i} \left(\boldsymbol{X}_{h,\mathcal{S}\cup\{j\}}\boldsymbol{w} - \boldsymbol{y}_h \right)^2 + \lambda \|\boldsymbol{w}\|_2^2.$$

Despite the use of leave-one-out based selection heuristic, the running time of Greedy RLS is analogous to that of GLS, it scales linearly with respect to the number of features selected, the total number of features and the number of examples.

The class of feature selection methods based on ℓ_1-norm regularization incorporates a wide variety of methods. Here we focus primarily on Lasso and the Elastic Net method, but other variations can be obtained simply by changing the loss function. The objective function that Lasso minimizes is [20]:

$$\sum_{i=1}^{m} (\boldsymbol{y}_i - \boldsymbol{X}_i\boldsymbol{w})^2 + \lambda \|\boldsymbol{w}\|_1 \ . \tag{8}$$

The number of features selected by Lasso and the running time of the algorithm are dependent on the value of the regularization parameter λ. The larger is the value, the smaller both the number of features being

selected and the number of iterations until convergence tend to be. However, unlike the methods directly optimizing the ℓ_0-norm, the dependence for Lasso is indirect in the sense that one can not tell exactly how many features will get selected with a given parameter value before training the model or how many iterations will be required, as both of them are very much dependent on the data. Similarly to GLS, Lasso is only good for selecting less than m features [20], which may be problematic in GWAS where there is often a small effect size for the individual variants and large numbers of SNPs may be necessary to produce suitable models. The use of cross-validation for selecting the value of λ can make the problem even worse, since part of the data must be reserved for validating the parameter values.

The method known as Elastic Net avoids the above-described drawback of Lasso to some extent. It represents a continuum between the Lasso and ridge regression methods, with the method acting as Lasso when $\lambda_1 > 0$ and $\lambda_2 = 0$ and as ridge regression when $\lambda_1 = 0$ and $\lambda_2 > 0$. In (9), the quadratic component removes the limitation of the number of selected variables with Lasso, it encourages grouping and helps to stabilize the ℓ_1 regularization [20]. As mentioned in [20], this means that the Elastic Net is a more viable solution for methods making use of grouping effects. By grouping we mean the effect of correlated features having a similar effect on the model, which might be a useful method in applications such as in pathway analysis. Zou et al. calls the equation $(1-\alpha)\|\boldsymbol{w}\|_1 + \alpha\|\boldsymbol{w}\|_2^2$ the Elastic Net penalty, where $\alpha = \lambda_2/(\lambda_2+\lambda_1)$ [20].

$$\sum_{i=1}^{m} (\boldsymbol{y}_i - \boldsymbol{X}_i\boldsymbol{w})^2 + \lambda_2\|\boldsymbol{w}\|_2^2 + \lambda_1\|\boldsymbol{w}\|_1 \tag{9}$$

Having the capability to fluctuate between Lasso and ridge regression affords the opportunity to provide both sparse solutions while, allowing for the shrinkage necessary for the model to not overfit the training data. Additionally, as it can be trained through the use of efficient optimization techniques such as coordinate descent, it potentially does not suffer from as high run times as wrapper methods. This does not mean that Elastic Net will not lead to universally better results than other leading algorithms, as the suitability of the method can depend on the applied search function and the dataset used.

Method Implementations for the Experiments

In order to examine their practical performance on common datasets, representative examples of machine learning methods were implemented on two SNP studies. The first experiment used WTCCC-T1D cases, combined with the UK National Blood Service and the 1958 Birth Cohort's control sets [27]. We implemented quality control procedures to filter based on having a MAF (5%), missing rate (1%), HWE (0.001), the genotype quality score (Chiamo value of 0.9) and exclusion lists provided by the WTCCC. This resulted in a set of 1,915 cases and 2,871 controls. The genotypes were encoded in the prediction models as binary genetic features through a genotype model which transforms each minor allele dosage (represented by 0,1,2) into three binary features: 0→(1,0,0), 1→ (0,1,0), 2→ (0,0,1), NA→ (0,0,0). This resulted in 986,331 binary genetic features in the T1D case, tripling the dataset size and memory requirements, while allowing the models to deal with missing genotype data (NA).

The second experiment involved a genetic cross between two yeast strains (Y2C), originally used to estimate the effect of epistasis on missing heritability of various quantitative yeast traits under highly-controlled laboratory conditions [28]. This dataset consisted of 1,008 segregants, 30,594 SNPs and 46 traits. We randomly selected one of the traits (Tunicamycin), but note that the Y2C data could be used to model genetic interactions also across multiple quantitative traits (pleiotropy, see Discussion). The only quality control procedure that we implemented was to remove those individuals with missing values for the particular trait. The original genotype data was adjusted so that variants sharing the same genotype across all of the segregants were merged, resulting in a set of 11,623 genetic features. Since the haploid dataset contains only two genotypic values (R and B), the genotypes were encoded by binary features (1 and 0).

For the case-control T1D data, we implemented a standard χ^2–based filter, by first selecting the top 500 variants according to their association p-values for each of the external folds, followed by L_2-regularized (ridge) regression. For wrappers, we used our greedy L_2-regularized least squares (RLS) implementation [1],

while for embedded methods, Lasso, Elastic Net and L_1-penalized logistic regression, were implemented through the Scikit-Learn package [29]. As a baseline reference method, we used the log odds-ratio weighted polygenic model [30], implemented as a weighted sum of the minor allele dosage in the 500 selected variants within each individual. For the quantitative Y2C data, we compared the performance of the greedy RLS, Lasso and Elastic Nets to a filter method, which selected the top 1,000 variants based on R^2, and then optimized the L_2-regularization parameter for RLS using nested CV. As a baseline method, we implemented a greedy version of least squares (LS), as this represents a model that is theoretically similar to the stepwise forward regression used in the original work [28]; greedy LS differs from the greedy RLS in terms that it implements regularization through optimization of L_0 norm instead of L_2 norm used in RLS.

Due to the large amount of processing power and memory needed for performing GWAS-scale experiments, a supercomputer at CSC - Finland's IT Center for Computer Science was used. The Hippu server is composed of a pair of HP ProLiant DL580 G7's and a pair of HP ProLiant DL785 G5s. The machine has an Rpeak of 1280 Gflop/s, and the two G7 servers have a total of 2 TB of memory while the two G5 servers have a total of 1 TB of memory. Additionally, the G7s are equipped with a total of eight 8-core Intel Xeon processors and the G5's have a total of 16 quad-core AMD Opteron processors.

As expected, filters have the lowest running times as they simply calculate a test statistic over all features. While greedy RLS tends to be fast when selecting a small amount of features, the cyclic coordinate descent based Lasso and Elastic Net implementations are more efficient when selecting a large amount of features. The main computational bottleneck, however, results from the need to select the hyperparameters of the learners. Selecting the ℓ_2-regularization parameter for greedy RLS and Elastic Net requires training the methods K-times for each tested parameter value, when K-fold cross-validation is used to select the parameter value. Further, when using a ℓ_1-regularizer for controlling the amount of selected features, such as is the case for Lasso and Elastic Net, a grid of parameters needs to be tested. Finally, if we do not have separate test sets, selecting parameters and evaluating test performance requires nested cross-validation, where inner cross-validation is used for parameter selection and outer-cv for performance evaluation. Combining nested cross-validation with parameter grid searches results in a combinatorial explosion that results in running times that are measured in days (e.g. Lasso), or weeks (Elastic Net and greedy RLS). This problem can be alleviated by using smaller or sparser parameter grids, small amount of folds or simpler heuristics for parameter selection. For example, for greedy RLS one may estimate the regularization parameters based on a filtered subset of the data and still provide a reasonable estimate. This allows selecting the hyperparameters in a matter of minutes.

Computational Validation of Predictive Accuracy

As the models become increasingly complex, their prediction errors decrease with the number of selected variants and other model parameters, which capture increasing details of the training data. However, this is true only to a certain extent for independent test data; while increasing complexity first allows for more accurate modeling, the test set error begins later to increase as the complexity of the model is no longer improving the generalization power [31]. Such model overfitting necessitates the use of a careful model validation, even after model regularization. Since the use of the same dataset during both the model construction and model validation may lead to a severe selection bias [32] resulting in overoptimistic estimates of predictive accuracy, separate validation data is needed.

A straightforward validation option is to apply the trained model onto an independent set of samples, which has not been examined during the whole model construction process. However, in addition to leading to a smaller proportion of training data, perhaps affecting the model generalizability, a within-study hold-out validation is prone to being effected by any experimental errors that may exist in the particular study. Between-study evaluation is a valid option in case such replication samples are available, especially if the model is intended to generalize beyond the genetic background of the training subjects; otherwise, population stratification methods may be needed to make the population structures more comparable [33].

Especially when limited numbers of samples are available, some type of cross-validation (CV) is frequently used to evaluate the predictive performance [1, 34]. In the simple K-fold CV, the sample is randomly

partitioned into K subsamples of equal size; the model is first trained on K-1 subsamples and then validated on the remaining sample (Figure 2). This process is repeated K times and an average over the K folds is used as an estimate of the predictive performance. Stratified CV guarantees that the phenotypic effect is similar in each fold; in disease classification, for instance, each fold contains approximately equal proportions of cases and controls. Further, when one needs to use CV both for parameter selection (including feature selection) and for estimating the accuracy of the learned model, the CV procedure should be nested. That is, on each round of CV (outer CV), where the data is split into a training set consisting of $K-1$ folds and the test set formed from the remaining fold, one performs also CV on this training set (inner CV) in order to select the learner parameters (see Figure 2). Such procedures can provide performance estimates free of a selection bias [31, 35]. After this estimate has been computed, the final model construction or feature selection can be performed on all the available data combined in order to use all the information available. In the two examples cases considered here the performance evaluation was implemented using nested 3-fold CV.

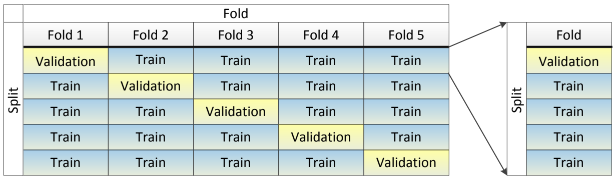

Figure 2: Organization of the standard cross-validation (left) and nested cross-validation (right) in terms of splitting the genetic data into the training and validation folds.

One of the most commonly reported metrics for quantifying the performance in the case-control setting is the area under the receiver operating characteristic curve (AUC). While having some specific caveats, the AUC has the advantage over many other metrics of being invariant to unbalanced settings, where the number of cases (m^+) and controls (m^-) is drastically different [1, 33, 36–38]. The AUC corresponds to the probability that the predicted phenotype of a randomly selected case ($\hat{y}_i^+$) will be ranked higher than that of a randomly selected control ($\hat{y}_j^-$). In its most basic formulation [39], the AUC can be summarized as

$$AUC = \frac{1}{m^+ m^-} \sum_{j=1}^{m^+} \sum_{k=1}^{m^-} g(\hat{\boldsymbol{y}}_j^+ - \hat{\boldsymbol{y}}_k^-) \tag{10}$$

where $g(x) = 0$, 0.5 and 1 if $x < 0$, $x = 0$ and $x > 0$, respectively. For an ideal classifier, AUC = 1, whereas a random classifier obtains an AUC = 0.5 on average. The AUC is closely related to the Mann-Whitney test statistic. While being useful in many applications, any single summary metric cannot capture all of the different tradeoffs in the predictive modeling. For instance, the true positive rate (sensitivity) is often more important in clinical applications than the false positive rate (1-specificity). The partial AUC can be used in such applications to integrate the sensitivity levels of a model up to a specified specificity cut-off.

In regression problems, the predictive accuracy for a continuous trait is often evaluated in terms of the coefficient of determination (R^2). This metric corresponds to the proportion of the phenotypic variance explained by the genetic model. Using squared errors between the observed y_i and predicted $\hat{y}_i$ phenotypes, R^2s is formally defined by the ratio of the variance accounted for by the model fitted to the training set relative to the variance of the phenotypic trait in the validation sample:

$$R^2 = 1 - \frac{\sum_{i=1}^{m} (\boldsymbol{y}_i - \hat{\boldsymbol{y}}_i)^2}{\sum_{i=1}^{m} (\boldsymbol{y}_i - \bar{\boldsymbol{y}})^2} \tag{11}$$

Here, $\bar{y}$ is the mean of the phenotypic trait over all the m individuals. Higher values of R^2 indicate larger portion of explained variance and hence a more predictive model. R^2 is also related to the estimated heritability (h^2), which corresponds to the proportion of phenotypic variance explained by true genetic values in the base population; however, since R^2 ignores inbreeding, relationships between individuals and estimation errors, it cannot be used as a consistent estimate of heritability [40].

References

1. Pahikkala T, Okser S, Airola A, Salakoski T, Aittokallio T: **Wrapper-based selection of genetic features in genome-wide association studies through fast matrix operations.** *Algorithms for Molecular Biology* 2012, **7**:11.
2. Okser S, Lehtimäki T, Elo LL, Mononen N, Peltonen N, Kähönen M, Juonala M, Fan YM, Hernesniemi JA, Laitinen T, Lyytikäinen LP, Rontu R, Eklund C, Hutri-Kähönen N, Taittonen L, Hurme M, Viikari JSA, Raitakari OT, Aittokallio T: **Genetic Variants and Their Interactions in the Prediction of Increased Pre-Clinical Carotid Atherosclerosis: The Cardiovascular Risk in Young Finns Study**. *PLoS Genetics* 2010, **6**(9):e1001146.
3. Wei Z, Wang K, Qu HQ, Zhang H, Bradfield J, Kim C, Frackleton E, Hou C, Glessner JT, Chiavacci R, Stanley C, Monos D, Grant SFA, Polychronakos C, Hakonarson H: **From Disease Association to Risk Assessment: An Optimistic View from Genome-Wide Association Studies on Type 1 Diabetes**. *PLoS Genetics* 2009, **5**(10):e1000678.
4. Kooperberg C, LeBlanc M, Obenchain V: **Risk prediction using genome-wide association studies**. *Genetic epidemiology* 2010, **34**(7):643–652.
5. Saeys Y, Inza I, Larrañaga P: **A review of feature selection techniques in bioinformatics**. *Bioinformatics* 2007, **23**(19):2507–2517.
6. Roshan U, Chikkagoudar S, Wei Z, Wang K, Hakonarson H: **Ranking causal variants and associated regions in genome-wide association studies by the support vector machine and random forest.** *Nucleic acids research* 2011, **39**(9).
7. Long N, Gianola D, Rosa GJM, Weigel KA, Avendano S: **Machine learning classification procedure for selecting SNPs genomic selection: application to early mortality in broilers**. *Journal of Animal Breeding and Genetics* 2007, **124**(6):377–389.
8. Purcell S, Neale B, Todd-Brown K, Thomas L, Ferreira MA, Bender D, Maller J, Sklar P, de Bakker PI, Daly MJ, Sham PC: **PLINK: a tool set for whole-genome association and population-based linkage analyses**. *American journal of human genetics* 2007, **81**(3):559–575.
9. Raymond M, Rousset F: **An Exact Test for Population Differentiation**. *Evolution* 1995, **49**(6):1280–1283.
10. Szumilas M: **Explaining odds ratios**. *Journal of the Canadian Academy of Child and Adolescent Psychiatry* 2010, **19**(3):227–229.
11. Pati Y, Rezaiifar R, Krishnaprasad PS: **Orthogonal Matching Pursuit: Recursive Function Approximation with Applications to Wavelet Decomposition**. In *Proceedings of the 27th Annual Asilomar Conference on Signals, Systems, and Computers* 1993:40–44.
12. Pahikkala T, Airola A, Salakoski T: **Speeding up Greedy Forward Selection for Regularized Least-Squares**. In *Proceedings of The Ninth International Conference on Machine Learning and Applications (ICMLA'10)*. Edited by Draghici S, Khoshgoftaar TM, Palade V, Pedrycz W, Wani MA, Zhu X, IEEE Computer Society 2010.
13. Nocedal J, Wright SJ: *Numerical Optimization*. Springer 2000.
14. Shi G, Boerwinkle E, Morrison AC, Gu CC, Chakravarti A, Rao DC: **Mining gold dust under the genome wide significance level: a two-stage approach to analysis of GWAS.** *Genetic epidemiology* 2011, **35**(2):111–118.
15. Srivastava S, Chen L: **Comparison between the stochastic search variable selection and the least absolute shrinkage and selection operator for genome-wide association studies of rheumatoid arthritis.** *BMC Proceedings* 2009, **3**(Suppl 7):S21.
16. González-Recio O, de Maturana EL, Vega AT, Engelman CD, Broman KW: **Detecting single-nucleotide polymorphism by single-nucleotide polymorphism interactions in rheumatoid arthritis using a two-step approach with machine learning and a Bayesian threshold least absolute shrinkage and selection operator (LASSO) model.** *BMC Proceedings* 2009, **3**(Suppl 7):S63.
17. Wei Z, Wang W, Bradfield J, Li J, Cardinale C, Frackelton E, Kim C, Mentch F, Van Steen K, Visscher PM, Baldassano RN, Hakonarson H: **Large Sample Size, Wide Variant Spectrum, and Advanced Machine-Learning Technique Boost Risk Prediction for Inflammatory Bowel Disease**. *American journal of human genetics* 2013, **92**(6):1008–1012.
18. Li J, Das K, Fu G, Li R, Wu R: **The Bayesian lasso for genome-wide association studies**. *Bioinformatics* 2011, **27**(4):516–523.

19. Tibshirani R: **Regression Shrinkage and Selection Via the Lasso**. *Journal of the Royal Statistical Society, Series B* 1994, **58**:267–288.

20. Zou H, Hastie T: **Regularization and Variable Selection via the Elastic Net**. *Journal of the Royal Statistical Society: Series B (Statistical Methodology)* 2003, **67**(2):301–320.

21. Ng AY: **Feature Selection, L1 vs. L2 Regularization, and Rotational Invariance**. In *Proceedings of the Twenty-first International Conference on Machine Learning*, ICML '04 2004:78–85.

22. Park MY, Hastie T: **Penalized logistic regression for detecting gene interactions**. *Biostatistics* 2008, **9**:30–50.

23. Zhu J, Rosset S, Tibshirani R, Hastie TJ: **1-norm Support Vector Machines**. In *Advances in Neural Information Processing Systems 16*. Edited by Thrun S, Saul L, Schölkopf B, MIT Press 2004:49–56.

24. Vapnik VN: *The nature of statistical learning theory*. New York, NY, USA: Springer-Verlag New York, Inc. 1995.

25. Chetty VK: **Ordinary Least Squares Regression**. In *Encyclopedia of Medical Decision Making*. Edited by Kattan MW, SAGE Publications, Inc. 2009:838–844.

26. Hoerl AE, Kennard RW: **Ridge Regression: Biased Estimation for Nonorthogonal Problems**. *Technometrics* 1970, **12**:55–67.

27. Burton PR, Clayton DG, Cardon LR, Craddock N, Deloukas P, Duncanson A, Kwiatkowski DP, McCarthy MI, Ouwehand WH, Samani NJ, Todd JA, Donnelly P, Et Al: **Genome-wide association study of 14,000 cases of seven common diseases and 3,000 shared controls**. *Nature* 2007, **447**:661–678.

28. Bloom JS, Ehrenreich IM, Loo W, Lite TLV, Kruglyak L: **Finding the sources of missing heritability in a yeast cross**. *Nature* 2013, **494**(7436):234–237.

29. Pedregosa F, Varoquaux G, Gramfort A, Michel V, Thirion B, Grisel O, Blondel M, Prettenhofer P, Weiss R, Dubourg V, Vanderplas J, Passos A, Cournapeau D, Brucher M, Perrot M, Duchesnay E: **Scikit-learn: Machine Learning in Python**. *Journal of Machine Learning Research* 2011, **12**:2825–2830.

30. Evans DM, Visscher PM, Wray NR: **Harnessing the information contained within genome-wide association studies to improve individual prediction of complex disease risk.** *Human molecular genetics* 2009, **18**(18):3525–3531.

31. Okser S, Pahikkala T, Aittokallio T: **Genetic variants and their interactions in disease risk prediction - machine learning and network perspectives.** *BioData mining* 2013, **6**:5.

32. Ambroise C, McLachlan GJ: **Selection bias in gene extraction on the basis of microarray gene-expression data**. *Proceedings of the National Academy of Sciences of the United States of America* 2002, **99**(10):6562–6566.

33. Wray NR, Yang J, Hayes BJ, Price AL, Goddard ME, Visscher PM: **Pitfalls of predicting complex traits from SNPs.** *Nature reviews. Genetics* 2013, **14**(7):507–515.

34. Kruppa J, Ziegler A, König I: **Risk estimation and risk prediction using machine-learning methods.** *Human genetics* 2012, **131**(10):1639–1654.

35. Varma S, Simon R: **Bias in error estimation when using cross-validation for model selection**. *BMC Bioinformatics* 2006, **7**(91).

36. Jostins L, Barrett JC: **Genetic risk prediction in complex disease.** *Human Molecular Genetics* 2011, **20**(R2):R182–8.

37. Jakobsdottir J, Gorin MB, Conley YP, Ferrell RE, Weeks DE: **Interpretation of Genetic Association Studies: Markers with Replicated Highly Significant Odds Ratios May Be Poor Classifiers**. *PLoS Genetics* 2009, **5**(2):e1000337.

38. Janssens A, van Duijn CM: **Genome-based prediction of common diseases: methodological considerations for future research.** *Genome Medicine* 2009, **1**(20).

39. Hanley JA, McNeil BJ: **The meaning and use of the area under a receiver operating characteristic (ROC) curve**. *Radiology* 1982, **143**:29–36.

40. Makowsky R, Pajewski NM, Klimentidis YC, Vazquez AI, Duarte CW, Allison DB, de los Campos G: **Beyond Missing Heritability: Prediction of Complex Traits**. *PLoS Genetics* 2011, **7**(4):e1002051.
